# Tone burst evoked otoacoustic emissions in neonates

**DOI:** 10.1016/S1808-8694(15)30664-9

**Published:** 2015-10-19

**Authors:** Jordana Costa Soares, Renata Mota Mamede Carvallo

**Affiliations:** 1 MSc in Sciences at Faculdade de Medicina USP, Speech and Hearing Therapist; 2 PhD, Professor at the Speech and Hearing Therapy Program at FMUSP

**Keywords:** spontaneous otoacoustic emissions, neonate, hearing tests, neonatal screening

## Abstract

A potential research project in otoacoustic emissions is the use tone bursts - frequency-specific stimulus.

**Aim:**

to study otoacoustic emission responses evoked by tone bursts in neonates with hearing loss risk factors.

**Materials and Methods:**

21 neonates with risk factors for hearing loss (study group) and 30 neonates without these risk factors (control group) were evaluated by otoacoustic emissions at the frequency range of 2,000 and 4,000 hertz.

**Study:**

Clinical and experimental.

**Results:**

There was a right ear advantage in female individuals and in the control group, although without statistical significance. Mean “Response” values at 2,000 hertz were 17.73 dB in the control group and 16.55 dB in the study group for female subjects; and 16.63 dB in the control group and 16.12 dB in the study group for male subjects. At 4,000 hertz, the values were 14.63 dB in the control group and 15.09 dB in the study group for female subjects; and 18.57 dB in the control group and 15.06 dB in the study group for male subjects.

**Conclusion:**

Tone bursts may help evaluate cochlear function in neonates.

## INTRODUCTION

Otoacoustic emissions (OAE) are sounds generated by the outer hair cells (OHC) inside a normal cochlea in response to acoustic stimulation. Part of this sound returns from the cochlea, passes through the tympanic-ossicular system, and arrives at the ear canal to be captured by a miniature microphone^1^,^2^.

OAE tests are extremely useful in screening patients for hearing and are a valuable diagnostic tool. The test is quick, non-invasive, objective, sensitive, frequency-specific, and can be performed in non-soundproofed facilities. It can also be quite useful in delivering differential diagnosis, monitoring therapy, selecting between hearing aids and surgical procedures, but is no substitute for tone threshold audiometry^3^,^4^.

A great deal of the OAE tests use linear and non-linear 'clicks', both known for having broad frequency ranges. The use of stimulation at specific frequencies to improve audiological diagnosis has been targeted by studies looking at brainstem auditory evoked potential (BAEP)^5^, ^6^, ^7^, ^8^. However, only a few studies have used tone bursts (TB) to measure OAEs in neonates.

Tone bursts allow for more specific hearing tests per frequency when compared to stimulation by clicks. TB stimulation also provides enhanced concentration of the energy to be applied in the cochlea without reaching the non-linear overload region of the probes. In other words, the increased intensity peak is traded for duration. Outside the more active acoustic frequency band (1-2 kHz), responses can be elicited by TB stimulation in an area where click stimulation would struggle^3^.

Responses to TBEOAE were found at lower frequencies (0.5 and 1 kHz) in individuals without and with sensorineural hearing loss, although in the latter group the TBEOAEs were of a lower magnitude^9^.

On a TBEOAE study done in animals, the authors concluded that OAEs can be affected by metabolic changes in the hair cells, and that this type of stimulation can be useful in assessing histological and functional hair cell damage^10^.

High levels of reliability were found in TBEOAE responses one day after the first test and one month later in young adults with audiometric threshold within normal ranges. Responses were more reliable and frequent at 1.5 and 2 kHz. At high and medium intensities, responses presented greater amplitude and reproducibility when compared to individual frequency bands in click stimulation tests. The authors indicated that TB is potentially useful in clinical evaluation^11^.

In a neonatal screening program using TB stimulation, more neonates were found to have OAEs, thus reducing the need for retesting. The authors proposed that TB stimulation is used to supplement neonatal hearing screening^12^.

Only a very few studies have been done with frequency-specific TB stimulation to measure OAEs, and even fewer in neonate populations. This paper aims to analyze the magnitude of OAE responses evoked by TB stimulation at specific frequencies in neonates with and without risk factors for hearing loss, thus increasing the amount of information on cochlear hearing function in this population.

## OBJECTIVE

This study looked at the magnitude of the OAE responses in neonates with and without risk factors for hearing loss under stimulation at 2000 and 4000 Hz. More specifically, the following indicators were targeted:

• Response variation between right and left ears
• Response variation between males and females
• Response variation between control and case groups

## MATERIALS AND MÉTHOD

Sample

The sample was made up of 51 full term neonates analyzed between January and June of 2007, distributed in the following fashion:

• Control group: 30 neonates, 15 females and 15 males without risk factors for hearing loss13.
• Case group: 21 neonates, 12 females and 9 males with at least one risk factor for hearing loss13.

Given the relationship between consanguinity and hearing loss shown in some publications in the literature14-17, this factor was also utilized in our study.

Equipment

• ILO 88 / ECHOPORT PLUS Otodynamics Analyzer
• Laptop computer, Pentium III processor with color monitor with ILO V5.6 Echoport Plus Otodynamics Analyzer installed.
• Neonatal probe (SNS-8) connected to channel A on the equipment's external unit.

### Procedure

This study was approved by the Research Ethics Committee at the Hospital Universitário da Universidade de São Paulo under permit 607/05, and by the Ethics Committee for Research Project Analysis (CAPPesq) at the Hospital das Clínicas Faculdade de Medicina da Universidade de São Paulo under permit 176/06. The parents of the neonates were informed of the objectives of the study and, upon their agreement, they were asked to sign a free informed consent form. A file for each neonate was then prepared, containing prenatal and neonatal health data and family aspects connected to communication (hearing and speech).

Later on, the neonates - preferably when they were asleep - were taken in their cribs to a quiet, non-soundproofed room adjacent to the nursery for assessment. OAE acquisition was performed between 36 hours and 28 days of age for both groups. Half of the tests were initiated by the right ear while the other half by the left ear.

### Neonatal hearing screening

The first test performed was OAE acquisition, on 'quickscreen' mode. Response analysis time is 12 ms. Clicks were used for stimulation with duration of 80 ms and intensity ranging between 78 and 83 dB peak equivalent. This test was performed to make sure subjects met the enrollment criteria looking at presence of OAEs in screening mode.

### Tone-burst-evoked otoacoustic emissions

After screening, TBEOAE acquisition began for 2 and 4 kHz, separately, at intensities ranging between 60 and 65 dB peak equivalent. Response analysis time is 20 ms. At the end of the tests, variables 'Response' (overall response), 'A&B mean' (mean wave intensity), and specific frequency band response (2 and 4 kHz) were considered for statistical analysis.

### Statistical method

The data sets were analyzed using the non-parametric Mann-Whitney test. Adding to the descriptive analysis, a Confidence Interval value was applied for mean values. A significance level of 0.05 (5%) was adopted. All confidence intervals were built with 95% statistical confidence. Significant differences were marked with an asterisk (*).

## RESULTS

### Sample characterization

The case group had at least one risk factor for hearing loss. Risk factor occurrence can be seen on Table 1.

**Table 1:** Hearing loss risk factor occurrence.

Risk factor	Occurrence (n=21)
Use of ototoxic antibiotics	7 (33,3%)
Consanguinity	4 (19%)
Low Apgar score	3 (14,2%)
Family history of hearing loss (FHHL)	3 (14,2%)
Gestational infection (toxoplasmosis)	1 (4,8%)
Malformation (auricular appendix)	1 (4,8%)
Malformation (auricular appendix) and FHHL	1 (4,8%)
Hyperbilirubinemia	1 (4,8%)

TBEOAE response magnitude and comparative study

The comparison between right and left ears for each of the genders, response type (A&B Mean, Response, and band specific response) within the groups with 2 and 4 kHz stimulation did not reveal statistically significant differences in any of the tests, as seen in Table 2, Table 3. No statistically significant differences were found between the responses from the case and control groups, as seen in Table 4.

**Table 2:** Response magnitude (dB), descriptive analysis and comparison (p-value) between RE and LE for control group, on TB 2 kHz and TB 4 kHz.

TB 2kHz										
	Control group		Mean	Median	St. Dev.	Q1	Q3	N	CI	p-value
		D	19,19	18,50	3,53	17,65	21,45	7	2,62	
	A&B Mean									0,247
		E	16,04	17,40	4,99	11,05	20,33	8	3,46	
		D	18,82	18,85	4,76	17,80	22,08	6	3,81	
Female	Response									0,855
		E	19,24	20,30	2,20	17,50	20,30	5	1,93	
		D	14,00	14,00	7,37	11,00	19,00	7	5,46	
2kHz										0,558
		E	7,43	7,00	7,98	1,50	10,50	7	5,91	
		D	14,00	11,85	5,37	9,83	18,88	8	3,72	
	A&B Mean									0,417
		E	15,37	15,90	3,36	12,70	17,95	7	2,49	
		D	16,86	17,80	7,50	11,95	22,70	7	5,56	
Male	Response									0,685
		E	15,38	15,90	4,36	14,90	18,40	5	3,82	
		D	11,38	10,00	9,33	6,50	17,50	8	6,47	
	2kHz									0,954
TB 4kHz										
	Control group		Mean	Median	St. Dev.	Q1	Q3	N	CI	p-value
		D	16,27	15,80	3,56	14,10	19,20	7	2,63	
	A&B Mean									0,062#
		E	12,69	11,40	4,48	10,73	12,65	8	3,11	
		D	16,45	16,85	3,52	14,65	18,98	6	2,82	
Female	Response									0,100
		E	12,68	10,30	5,96	10,10	10,50	5	5,22	
		D	14,83	15,50	6,11	13,50	190,0	6	4,89	
		4 kHz								0,086#
		E	10,00	14,00	10,20	-1,25	19,00	8	7,07	
		D	17,75	16,65	5,66	14,63	21,43	8	3,92	
	A&B Mean									0,728
		E	15,16	14,90	5,77	12,35	17,90	7	4,28	
		D	17,15	18,15	4,86	15,73	19,58	4	4,77	
Male	Response									1,000
		E	18,13	17,35	4,77	15,83	19,65	4	4,67	
		D	10,14	6,00	8,67	3,50	15,50	7	6,42	
	4 kHz									0,745
		E	11,40	9,00	11,01	4,00	19,00	5	9,65

**Table 3:** Response magnitude (dB), descriptive analysis and comparison (p-value) between RE and LE for case group, on TB 2 kHz and TB 4 kHz.

TB 2kHz										
	Control group		Mean	Median	St. Dev.	Q1	Q3	N	CI	p-value
		D	17,07	17,25	5,02	13,73	20,48	6	4,02	
	A&B Mean									0,423
		E	15,32	13,20	5,21	12,38	16,80	6	4,17	
		D	17,84	19,30	4,79	13,70	20,60	5	4,20	
Female	Response									0,251
		E	14,96	11,40	6,14	10,70	17,40	5	5,38	
		D	7,83	8,00	9,35	1,75	12,00	6	7,48	
	2kHz									0,687
		E	10,83	10,00	6,97	7,75	14,50	6	5,58	
		D	14,04	12,40	4,13	10,50	17,30	5	3,62	
	A&B Mean									0,140
		E	18,83	18,65	2,70	17,88	19,60	4	2,65	
		D	13,58	14,25	5,53	10,45	17,38	4	5,42	
Male	Response									0,248
		E	18,15	18,35	3,56	17,13	19,38	4	3,49	
		D	6,80	10,00	6,42	2,00	12,00	5	5,63	
	2kHz									0,085#
		E	15,75	17,00	5,56	14,00	18,75	4	5,45	
TB 4kHz										
	Control group		Mean	Median	St. Dev.	Q1	Q3	N	CI	p-value
		D	13,97	14,90	4,12	10,88	16,00	6	3,30	
	A&B Mean									0,631
		E	14,82	12,70	7,56	10,43	14,23	6	6,05	
		D	13,26	14,20	5,01	11,50	15,60	5	4,39	
Female	Response									0,655
		E	18,23	13,10	9,59	12,70	21,20	3	10,85	
		D	8,67	9,50	9,54	1,00	15,75	6	7,64	
	4 kHz									0,272
		E	4,20	2,00	7,43	-1,00	12,00	5	6,51	
		D	14,30	14,40	5,54	10,30	14,80	5	4,86	
	A&B Mean									0,624
		E	14,53	14,10	3,31	12,53	16,10	4	3,24	
		D	16,53	13,30	5,60	13,30	18,15	3	6,34	
Male	Response									0,554
		E	16,40	16,40	2,69	15,45	17,35	2	3,72	
		D	9,20	14,00	11,61	-1,00	16,00	5	10,17	
	4 kHz									0,459
		E	5,00	5,00	9,52	-2,00	12,00	4	9,33

**Table 4:** Response magnitude (dB), descriptive analysis and comparison (p-value) between control and case groups, for TB 2 kHz and TB 4 kHz

	2 kHz		Mean	Median	St. Dev.	Q1	Q3	N	CI	p-value
	A&B	Control	16,99	17,15	3,90	13,98	20,23	30	1,39	
		Case	16,90	16,75	4,56	13,20	20,45	24	1,82	0,807
		Control	17,73	17,70	3,81	15,30	20,55	23	1,56	
Female	Response									0,382
		Case	16,55	16,65	5,15	12,28	20,48	22	2,15	
		Control	12,14	14,00	7,95	9,00	19,00	29	2,89	
	2 kHz									0,449
		Case	10,96	12,00	7,15	6,50	16,00	23	2,92	
		Control	16,44	16,10	4,52	13,05	19,35	30	1,62	
	A&B									0,639
		Case	15,84	15,75	4,36	12,40	18,58	18	2,01	
		Control	16,63	17,30	5,37	14,45	19,70	23	2,19	
Male	Response									0,605
		Case	16,12	16,80	4,86	13,73	18,38	14	2,55	
		Control	11,46	11,50	7,98	7,00	16,75	28	2,96	
	2 kHz									0,628
		Case	10,11	12,00	7,30	6,00	15,75	18	3,37	
	4kHz		Mean	Median	St. Dev.	Q1	Q3	N	CI	p-value
		Control	14,52	12,95	3,94	11,50	18,80	30	1,41	
	A&B									0,741
		Case	14,34	13,75	4,85	10,93	16,25	24	1,94	
		Control	14,63	13,05	4,52	10,65	18,98	22	1,89	
Female	Response									0,567
		Case	15,09	14,20	5,64	11,65	18,25	15	2,86	
		Control	10,22	10,00	7,55	4,50	15,50	27	2,85	
	4 kHz									0,376
		Case	8,09	4,50	8,66	2,25	15,75	22	3,62	
		Control	14,76	13,90	5,50	10,40	19,10	30	1,97	
	A&B									0,840
		Case	14,04	12,90	4,15	11,13	15,13	18	1,92	
		Control	18,57	18,80	4,29	17,05	21,05	15	2,17	
Male	Response									0,165
		Case	15,06	13,90	5,25	13,30	19,58	10	3,25	
		Control	11,08	14,00	9,54	3,00	19,00	25	3,74	
	4 kHz									0,124
		Case	6,94	6,00	8,76	-1,00	15,00	17	4,17	

In gender comparison for the 2 kHz frequency, females had higher response levels for all analyzed variables in both case and control groups, although without statistically significant differences. For the 4 kHz frequency, females also presented higher response levels for all analyzed variables in the case group, yet without statistically significant difference. In the control group, however, the male subjects had higher response levels with statistically significant difference only on variable 'Response.' Response magnitude and comparative analysis can be seen in Table 5.

**Table 5:** Response magnitude (dB) and comparison (p-value) between genders.

TB 2 kHz	Response		A&B Mean		2 kHz	
	Control	Case	Control	Case	Control	Case
Female	17,73	16,55	16,99	16,90	12,14	10,96
Male	16,63	16,12	16,44	15,84	11,46	10,11
p-value	0,448	0,820	0,515	0,469	0,643	0,762
TB 4 kHz	Response		A&B Mean		4 kHz	
	Control	Case	Control	Case	Control	Case
Female	14,63	15,09	14,52	14,34	10,22	8,09
Male	18,57	15,06	14,76	14,04	11,08	6,94
p-value for genders	0,026*	0,781	0,882	0,959	0,783	0,561

## DISCUSSION

Use of ototoxic antibiotics topped the list of risk factors for hearing loss in terms of prevalence (33%), as also seen in other papers at however different percentages^18^,^19^. Parental consanguinity is not a frequently seen factor in neonatal screening studies, but it ranked second (19%) in our population. A number of papers, including some using genetic tests, discuss the relationship between consanguinity and hearing loss^14^, ^15^, ^16^, ^17^.

Low Apgar score, the third most prevalent risk factor, was observed in 14.2% of the neonates, as seen in the literature^20^. Family history of hearing loss (FHHL) tied at third (14.2%) and is a frequently reported factor, although not as highly prevalent in other papers^18^,^19^,^21^,^22^.

Lastly, risk factors congenital infection (toxoplasmosis), auricular appendix, hyperbilirubinemia, and auricular appendix combined with FHHL accounted each for 4.8%. These factors are reported in the literature with different prevalence rates^18^,^19^,^22^,^23^.

In terms of OAE magnitude, in both groups the 'Response' values for the two frequency bands were larger than the values obtained from click stimulation reported by Basseto^24^ – 13.5 dB for females and 13 dB for males - and Basseto et al.^25^ – 13.8 dB for right ears and 13.3 dB for left ears of females and 13.5 dB for right ears and 12.5 dB for left ears of males. However, Durante et al.^26^ found increased OAE response levels with click stimulation among both females (21.6 dB) and males (19.9 dB). In the only paper we found on neonate TBEOAE, the mean response values were 13.8 dB at 1.5 kHz, 17.5 dB at 2.2 kHz, and 17.4 dB at 3 kHz^12^.

Several authors have reported higher magnitudes of click-evoked OAE in right ears and females^24^,^26^, ^27^, ^28^. In a way, the findings described in this paper match the literature, as higher values were found for right ears at 2 kHz and 4 kHz, although without statistical significance.

It is known that predominantly crossed medial olivocochlear system stimulation in the brainstem from contralateral auditory stimulation leads to reduced OAE magnitude. Such OAE suppression effect is also present in neonates^29^, ^30^, ^31^, ^32^, ^33^. Increased suppression effect has also been found in right ears^29^,^33^, ^34^, ^35^. This same effect could grant right ears increased OAE response. Increased right ear click-evoked OAE responses are assigned to sound processing at the level of the cochlea and the brainstem, possibly facilitating further hemispheric specialized development for the processing of certain sound types36. Such specialization is attributed to the left auditory cortex^37^.

Although lacking statistical significance, females presented greater response magnitudes in all analyzed variables in both control and case groups at 2 kHz. Females kept on presenting greater response levels at 4 kHz in the case group, but in the control group responses were more discrete. Higher response levels in females may be associated with the gender's shorter cochlear length. In spite of a few differences, some authors have found shorter cochlear length in females^38^,^39^. In shorter cochleae, acoustic stimulation could get to the OHC more quickly, losing less sound energy, consequently eliciting better responses.

The differences found between control and case groups were not statistically significant for any of the analyzed variables, at either of the frequencies or genders. However, when considering numeric values, the control group had higher responses than the case group, except for females in variable 'Response' at 4 kHz. This advantage of the control group over the case group has also been observed in tests done with click stimulation^33^. In another paper, individuals with high frequency hearing loss had lower responses to TB at 0.5 and 1 kHz than subjects without high frequency hearing loss, showing that this stimulation may be used to differentiate between groups^9^.

The relevance of this paper lies in the possibility of improving neonatal hearing screening procedures for the population in general - whether or not at risk for hearing loss - contributing with the identification of responses in specific areas of the cochlea. There is a growing concern over offering quick, objective, effective tests that include scanning for specific frequencies. Tone burst stimulation can thus be used to complement neonatal hearing screening^12^.

## CONCLUSIONS

Specific frequency stimulation can be offered to neonates and produce mean responses ranging between 10.11 and 17,73 dBSPL for TBEOAE at 2 kHz, and 6.94 and 18.57 dBSPL at 4 kHz. Although without statistically significant difference, higher values were observed for right ears, females, and in the control group in the comparison between ears, gender, and groups.
